# British Methods and Men of Science

**Published:** 1906-08-18

**Authors:** 


### British Methods and Men of Science.

Last week we had occasion to point out that the discovery of aniline dyes fifty years ago, so far as its commercial value was concerned, was overlooked by the English community. Foreigners compete successfully with Englishmen in these matters because science and scientists are recognised and encouraged abroad, whilst in England they are regarded for the most part as dreamers or maniacs, geniuses if you will who are sometimes amusing, always dogmatic, and frequently quarrelsome. England has often been described as the treasure island of the world, and the very fact of the prosperity and wealth of the nation has made it indifferent or blind to the proper application of adequate money to the encouragement of science and scientists, and in many other directions which in modern times make for the success of peoples. The whole of the working-class population of these islands is threatened with permanent disaster, owing to the unscientific methods which dominate much of our business management. If these unscientific methods are continued by the obstinacy of the British people for a few more years, they may rob the working classes of England's manufacturing power and supremacy, and so ruin the nation.

The *Times* of the 8th instant published a letter from Dr. Ray Lankester, certainly one of the most eminent scientists of the day, who has done invaluable service as director of the Natural History Department of the British Museum. Dr. Ray Lankester is fifty-nine years of age; he is full of energy and work, and for practical purposes the output and quality of his work were never probably more valuable than they are to-day, and will continue to be for at least ten years to come. The nation is, however, to be deprived of Dr. Lankester's services, and Dr. Lankester himself is to be deprived of his laboratory, and of all the necessary means requisite to the utilisation of his genius in the service of the nation and the world, because he is not personally popular, and by accepting his present office in the service of the people he has brought himself technically under a rule of the Civil Service which provides that the head of a department may call upon any officer in it to retire at the age of sixty upon such pension as he is entitled to under the regulations. Dr. Lankester entered the national service when his reputation was world-wide, being fifty-two years of age, so that, as the *Times* points out, when he reaches sixty next May a regulation intended to apply to men who have spent their lives in a Government office, decrees that his pension shall be £160, which the Treasury of its goodness may raise to £300.

In any other country but England a case like that of Dr. Lankester would have created considerable national excitement, secured the interest and support of the best business minds in the country, and have probably filled the newspapers. Although the present is what is known as the dead season, no further letters appeared in the *Times* for six days, and then the only communication on the subject was one from Professor John W. Judd, another distinguished man of science, who was appointed professor of geology in the Royal School of Mines in 1876, having previously been for some years in the employ of the Government under Matthew Arnold. For thirty years Professor Judd has spent his life in organising geological teaching, and has done his work so well that it has become a model which has been extensively followed by other teachers, by the colonies, and by foreign nations too. On reaching the age of sixty-five Professor Judd acceded to the request of the Board of Education to continue his services for another term, but being compelled by his doctor's advice, on the grounds of health, to discontinue his work this year after thirty-six years' service, he felt, no doubt, that he was entitled to a minimum pension of £600 a year, having been promised on appointment an increase of salary up to £1,400 a year. The Treasury has now informed Professor Judd that they can only grant him a pension of nearly £100 per annum less than the minimum in question. Yet the last thirty years have made geology in the commercial sense one of the most important branches of science, and no man can have rendered services of more practical value to the commercial community in this respect than Professor Judd by his teaching, his work, and his accessibility as a consultant and adviser to all interested in his subject, without fee or reward.

It will be claimed, no doubt, that Professor Ray Lankester is an impossible person owing to the idiosyncrasies of his character, which has given him an intellect strong enough to spot stupidity in a moment, and to render him indifferent to methods of conciliation when occasion has arisen for an expression of his views. During Professor Judd's whole career there is no instance of friction of any kind between himself and the Board of Education in thirty-six years. We must remember, too, in this connection that true genius is a mind of large general powers, accidentally determined in some particular direction. Lowell's definition that "talent is that which is in a man's power; genius, that in whose power a man is," must destroy the case of those who defend the illiberal treatment by the nation of the scientific worker because of his idiosyncrasies. A nation without genius would soon become a dull, coarse compound, without initiative, losing touch with material matters, until it gradually disappeared. Of all nations on the face of the earth the British nation needs men of genius to compensate for the limited area of the earth's surface which its people occupy, and to enable the people of these islands to maintain their supremacy in the world of nations. The nation cannot afford, therefore, to treat men of scientific genius as Professors Lankester and Judd are being treated by Treasury red tape at the moment.

As a matter of business, too, what stupidity and wrong are exhibited by these two cases! The records of the Treasury contain scores of instances of men who have rendered routine services for the most part, keeping well within the technical regulations which govern such matters, and so securing on retirement even more than a maximum pension. The ordinary policeman who enters the service at twenty years of age can, and as a matter of fact does, retire after twenty-five years' service, that is at forty-five years of age, on a pension equal to three-fifths of his pay on retirement. The man of science, having risen to the height of his powers at sixty years of age, may be compulsorily retired, and his usefulness destroyed at the caprice of enemies, or routine regulations, or he may be harshly treated, like Professor Judd, by the machine action of Treasury officials. We shall soon be paying in municipal and similar pensions a sum equivalent to the charges for the National Debt, say six millions a year, whilst a hundredth part of this sum would provide a princely amount for the adequate retiring allowances and the full encouragement of the genius of all the men of science which the British nation is ever likely to have the good fortune to possess. Yet the British people plume themselves on their business qualities, although in these days of competition business success in a national sense is almost wholly dependent upon scientific methods, which must have their origin in the genius of men of science. The end of the whole matter is that England must change its methods of treatment for men of science, or the British people must make up their minds to prepare with what grace they can for the ultimate funeral of the British nation.

